# Molecular characterization and protective efficacy of the microneme 2 protein from *Eimeria tenella*

**DOI:** 10.1051/parasite/2018061

**Published:** 2018-11-26

**Authors:** Ming Yan, Xiaoxia Cui, Qiping Zhao, Shunhai Zhu, Bing Huang, Lu Wang, Huanzhi Zhao, Guiling Liu, Zhihang Li, Hongyu Han, Hui Dong

**Affiliations:** 1 Key Laboratory of Animal Parasitology of Ministry of Agriculture, Shanghai Veterinary Research Institute, CAAS Shanghai 200241 PR China; 2 College of Life and Environment Sciences, Shanghai Normal University Shanghai 200234 PR China; 3 Qingdao Yebio Biological Engineering Co., Ltd Qingdao 266114 PR China

**Keywords:** Coccidiosis, *Eimeria*, microneme-2, EtMIC2, invasion, immunization

## Abstract

Microneme proteins play an important role in the adherence of apicomplexan parasites to host cells during the invasion process. In this study, the microneme 2 protein from the protozoan parasite *Eimeria tenella* (EtMIC2) was cloned, characterized, and its protective efficacy as a DNA vaccine investigated. The EtMIC2 gene, which codes for a 35.07 kDa protein in *E. tenella* sporulated oocysts, was cloned and recombinant EtMIC2 protein (rEtMIC2) was produced in an *Escherichia coli* expression system. Immunostaining with an anti-rEtMIC2 antibody showed that the EtMIC2 protein mainly localized in the anterior region and membrane of sporozoites, in the cytoplasm of first- and second-generation merozoites, and was strongly expressed during first-stage schizogony. In addition, incubation with specific antibodies against EtMIC2 was found to efficiently reduce the ability of *E. tenella* sporozoites to invade host cells. Furthermore, animal-challenge experiments demonstrated that immunization with pcDNA3.1(+)-EtMIC2 significantly increased average body weight gain, while decreasing the mean lesion score and oocyst output in chickens. Taken together, these results suggest that EtMIC2 plays an important role in parasite cell invasion and may be a viable candidate for the development of new vaccines against *E. tenella* infection in chickens.

## Abstract (fr)

Les protéines des micronèmes jouent un rôle important dans l’adhésion des parasites Apicomplexa aux cellules hôtes au cours du processus d’invasion. Dans cette étude, la protéine de micronème 2 du protozoaire parasite *Eimeria tenella* (EtMIC2) a été clonée, caractérisée et son efficacité protectrice en tant que vaccin à ADN a été étudiée. Le gène EtMIC2, qui code pour une protéine de 35.07 kDa dans des oocystes sporulés d’*E*. *tenella*, a été cloné et la protéine EtMIC2 recombinante (rEtMIC2) a été produite dans un système d’expression d’*Escherichia coli*. Une immuno-coloration avec un anticorps anti-rEtMIC2 a montré que la protéine EtMIC2 se localisait principalement dans la région antérieure et la membrane des sporozoïtes, dans le cytoplasme des mérozoïtes de première et de deuxième génération, et était fortement exprimée au cours du premier stade de schizogonie. De plus, une incubation avec des anticorps spécifiques contre EtMIC2 s’est avérée efficace pour réduire la capacité des sporozoïtes d’*E. tenella* à envahir les cellules hôtes. En outre, des expériences d’infestation ont montré que l’immunisation avec pcDNA3.1(+)-EtMIC2 augmentait de manière significative le gain de poids corporel moyen, tout en diminuant le score de lésion moyen et l’excrétion d’oocystes des poulets. Pris dans leur ensemble, ces résultats suggèrent qu’EtMIC2 joue un rôle important dans l’invasion de cellules parasitaires et pourrait constituer un candidat viable pour la mise au point de nouveaux vaccins contre l’infection à *E. tenella* chez les poulets.

## Introduction

Avian coccidiosis, a protozoan parasitic disease caused by the intracellular apicomplexan parasite, *Eimeria* spp., leads to heavy economic losses in the poultry industry worldwide [5]. It causes an estimated loss of more than $3 billion USD per annum due to production losses and veterinary prophylactic measures [1, 33]. Poultry farmers mainly rely on the use of “coccidiostat” in the feed to treat and/or prevent *Eimeria* infection. However, rigorous use of anticoccidial drugs has led to the development of drug-resistant *Eimeria* strains [4, 23]. The second most effective way to prevent coccidiosis is the use of live anticoccidial vaccines; however, until recently the use of these vaccines has been limited to broiler and layer breeders only due to the limited production, chances of virulence reversibility, and high cost [1, 31]. Therefore, there has been an increased effort to develop new control strategies for *Eimeria* infection that target multiple stages of the parasitic invasion process. One of these approaches is to block the invasion of *Eimeria* into intestinal epithelial cells to prevent coccidiosis.

*Eimeria* spp. belong to the apicomplexan parasites, possessing a characteristic apical complex consisting of micronemes, rhoptries, and structural elements such as the conoid, polar ring, and subpellicular microtubules [27]. Micronemes are small membrane-bounded organelles located immediately beneath the cell membrane, near the anterior end of the apical complex, and release numerous soluble and transmembrane proteins [41]. Previous studies have shown that the proteins secreted by micronemes are involved in multiple interactions between the parasite and the host cell, specifically in relation to motility, attachment, recognition, and penetration, and thus play a crucial role in the invasion process of apicomplexan parasites [2, 3, 11, 22, 25, 35, 39]. The *E. tenella* microneme-2 gene (EtMIC2) was first identified by Tomley et al. [39] and since then several studies have suggested that EtMIC2 has good immunogenicity and may be a good vaccine candidate [6, 29, 32, 36, 45, 47]. In this study, EtMIC2 was cloned, characterized, and its protective efficacy as a DNA vaccine investigated.

## Materials and methods

### Ethics Statement

Coccidia-free chickens and rabbits were used in this study. The protocol was approved by the Animal Care and Use committee of the Shanghai Veterinary Research Institute, Chinese Academy of Agricultural Sciences. The animals were provided with water and food *ad libitum*. At the end of the experiments, the animals were euthanized in strict accordance with the international standards for animal welfare.

### Experimental chickens, parasites, and cells

One-day-old yellow feathered broilers were kept in wire cages under coccidia-free conditions and provided with coccidiostat-free feed and water *ad libitum*. Periodic examination of *Eimeria* infection of the chickens was done by microscopic examination of feces. The chickens were moved to an animal containment facility prior to the challenge with virulent oocysts.

*Eimeria tenella* was isolated from Shanghai [10] and stored in the Key Laboratory of Animal Parasitology at the Ministry of Agriculture, Shanghai Veterinary Research Institute of the Chinese Academy of Agricultural Sciences. These parasites were maintained and propagated in two-week-old coccidia-free chickens, as previously described [40]. Sporulated oocysts were obtained and purified using standard procedures [8]. Sporozoites were obtained from cleaned sporulated oocysts with *in vitro* excystation, and were purified using chromatography over columns packed with nylon wool and DE-52 cellulose [9]. Second generation merozoites were collected and purified from the cecal mucosa at 112 h post-inoculation from chickens inoculated with 1 × 10^5^ sporulated oocysts [34].

The chicken embryo fibroblast cell line, DF-1, was maintained and cultured in Dulbecco’s modified Eagle’s medium (DMEM) (Invitrogen, Carlsbad, CA, USA) supplemented with 10% fetal bovine serum (FBS) at 37 °C, 5% CO_2_ [13].

### Cloning of the EtMIC2 gene

A total of 1.0 × 10^7^ sporulated oocysts of *E. tenella* were ground using a pre-chilled mortar and pestle. Total RNA was isolated from sporulated oocysts using TRIzol (Invitrogen), according to the manufacturer’s protocol. To avoid DNA contamination, the extracted RNA preparations were treated additionally with RNase-free DNase I (Takara, Dalian, China) for 30 min at 37 °C. DNase I was then inactivated by heating at 75 °C for 10 min. RNA was quantified with UV spectrophotometry (Eppendorf, Hamburg, Germany), and its integrity verified with electrophoresis using a 1% agarose denaturing formaldehyde-ethidium bromide (EtBr) gel. Complementary DNA (cDNA) was synthesized from the total RNA using an M-MLV Reverse Transcriptase kit (Invitrogen).

The complete coding region of the EtMIC2 gene was amplified using primers designed with Primer Premier version 5.0 software according to the mRNA sequence published in GenBank (accession number: AF111839.1). The primer sequences were as follows: forward primer, 5′-CG**GGATCC**GTTGCATTGCATAACCTCAT-3′; reverse primer, 5′-CCC**AAGCTT**CGTCACTCTGCTTGAACCT-3′, containing BamHI and HindIII restriction sites (bold), respectively. Amplification was performed by an initial reaction at 95 °C (5 min) followed by 30 cycles of 95 °C (30 s), 48 °C (30 s), 72 °C (1 min), and a final extension at 72 °C (10 min). After amplification, gel electrophoresis was carried out and targeted bands were selected, purified, and ligated to the pGEM-T easy cloning vector (Promega, Madison, USA), according to the manufacturer’s instructions. These vectors were transformed to TOP10 *Escherichia coli*. Positive colonies were picked from the plates and checked using enzymatic digestion using the previously inserted restriction sites, followed by sequence confirmation by Invitrogen Bio-tech (Shanghai Huajin Gene Bio-tech Co. Ltd., Shanghai, China). The analyses of the cDNA and amino acid sequences of EtMIC2 were carried out as previously described [14]. Briefly, the molecular mass was obtained using translate tool software at the ExPASy server of the Swiss Institute of Bioinformatics (http://www.expasy.org/tools/protparam.html). The signal peptide and transmembrane (TM) regions were predicted using SignalP (http://www.cbs.dtu.dk/services/SignalP/) and TMHMM (http://www.cbs.dtu.dk/services/TMHMM-2.0/), respectively.

### Expression and purification of recombinant EtMIC2

The EtMIC2 gene was then cloned into the pET32a(+) vector (Novagen, Merck KGaA, Darmstadt, Germany) using the EcoRI and HindIII sites. The recombinant plasmid was confirmed by DNA sequencing and transformed into the *E. coli* BL21 (DE3) (Promega) expression strain. The expression of rEtMIC2 in *E. coli* was induced using 0.8 mM isopropyl-β-D-thiogalactopyranoside (IPTG, Sigma-Aldrich, St. Louis, MO, USA) at 37 °C for 8 h. The induced bacterial cells were collected by centrifugation and sonicated on ice. The supernatant was collected, and the recombinant protein was purified using a His Bind Purification kit (Novagen). The purified protein was analyzed with 12% sodium dodecyl sulfate polyacrylamide gel electrophoresis and its concentration determined using a BCA protein assay kit (Novagen). The purified protein was then stored in aliquots at −20 °C until further use [7].

### Production of polyclonal sera against recombinant EtMIC2

Two-month-old rabbits were immunized subcutaneously with 0.2 mg of purified rEtMIC2 emulsified in an equal volume of Freund’s complete adjuvant (Sigma-Aldrich). This was followed by three booster injections with rEtMIC2 emulsified in an equal volume of Freund’s incomplete adjuvant (Sigma-Aldrich) at two-week intervals. Seven days after the final immunization, serum was separated from rabbit blood. Serum collected before immunization was used as the negative control. The anti-sera were then stored at −80 °C until further use.

### Immunolocalization of EtMIC2 in parasites with an indirect immunofluorescence assay

The localization of the EtMIC2 protein was investigated using an immunofluorescence assay (IFA), as previously described [14], with slight modifications. In brief, DF-1 cells (3 × 10^5^ cells per well) were seeded in six-well plates (Corning, Corning, NY, USA) with pre-coated sterile coverslips and cultured in complete medium (DMEM containing 10% FBS, 100 U/mL penicillin/streptomycin, 2 mM L-glutamine) at 37 °C in 5% CO_2_ for 24 h. Freshly excysted sporozoites were incubated for 1 h at 41 °C in complete medium. Then the sporozoites were added to adherent cells at a ratio of one sporozoite per cell. Infected DF-1 cells were collected at 72 h post infection for fixation. Sporozoites in infected DF-1 cells were washed with phosphate buffered saline (PBS) for 10 min, fixed in 2% paraformaldehyde for 20 min, permeabilized with 1% Triton-X in PBS for 15 min, and then blocked with 2% bovine serum albumin in PBS overnight at 4 °C. The coverslips were then incubated with a rabbit anti-rEtMIC2 antibody (1:100 dilution) for 1 h at 37 °C, and then further incubated for 1 h with goat anti-rabbit IgG fluorescein isothiocyanate (FITC)-conjugated antibody (1:500 dilution; Sigma-Aldrich) in a moist, dark chamber. Cell nuclei were labelled with 10 μg/mL 4, 6-diamidino-2-phenylindole (DAPI, Beyotime, Haimen, China) for 10 min. The coverslips were washed three times in PBS with 0.5% Triton-X 100 after every step. The coverslips were finally mounted on glass slides using 60 μL of Fluoromount Aqueous Mounting Medium (Sigma-Aldrich) and viewed under a fluorescence microscope (Nikon, Tokyo, Japan). Sporozoites and second-generation merozoites were prepared for immunofluorescence observation using the same method.

### Sporozoite invasion inhibition assay

The invasion inhibition assay was based on the observation that *E. tenella* sporozoites invade DF-1 cells [12, 13]. All antibodies were purified using Protein A+G agarose (Beyotime), according to the manufacturer’s instructions, and their concentration was determined using a BCA protein assay kit (Novagen). DF-1 cells (3 × 10^5^ cells per well) were seeded in 24-well plates and cultured in DMEM with 10% FBS at 37 °C in 5% CO_2_ for 24 h. Freshly isolated sporozoites were labelled with the dye carboxyfluorescein diacetate succinimidyl ester (CFSE-Molecular Probes, Beyotime), according to the manufacturer’s instructions, and incubated at 37 °C with either 100, 200, 300, or 400 μg/mL of purified IgG against rEtMIC2 for 2 h. The same quantity of purified IgG from naive rabbit sera was used as a control. The sporozoite–IgG mixture was centrifuged at 2000 × *g* for 5 min. The pellet was then resuspended in DMEM containing 10% FBS, penicillin (100 U/mL) and streptomycin (100 μg/mL). Following this, the pre-treated sporozoites were used to infect adherent DF-1 cells at a ratio of one sporozoite per cell and then cultured at 41 °C in 5% CO_2_ for 12 h. Cells were washed twice in sterile PBS and then trypsinized, collected, and analyzed with Cytomics FC500 flow cytometry (Beckman Coulter Inc., Fullerton, CA, USA). All assays were performed in triplicate. The percentage of infected cells in the presence or absence of the inhibitory antibody was used to calculate inhibition rates, as previously described [12].

### Construction of the pcDNA3.1(+)-EtMIC2 DNA vaccine and its expression *in vitro*

The EtMIC2 gene was cloned into the pcDNA3.1(+) vector using the BamHI/HindIII sites. The recombinant plasmid was then sequenced at Invitrogen Biotech (Shanghai, China) to confirm that the EtMIC2 gene was inserted into the correct open reading frame. The recombinant plasmid pcDNA3.1 (+)-EtMIC2, and the empty plasmid pcDNA3.1(+) were prepared as a DNA vaccine using the Qiagen EndoFree Plasmid Kit (Qiagen Biotech, Beijing, China), following the manufacturer’s instructions. The eluted product was dissolved in 10 mM Tris-HCl pH 8.0 and 1 mM EDTA (TE) buffer at 1 mg/mL and stored at −20 °C until required.

DF-1 cells were grown in six-well plates until a confluency of 80%–90%. The cells were then transfected with 4 μg of pcDNA3.1(+)-EtMIC2 or the empty plasmid pcDNA3.1(+) using Lipofectamine 2000 (Invitrogen), as per the manufacturer’s instructions. Briefly, the transfection reagent and recombinant plasmid were mixed (10 mL Lipofectamine 2000 and 4 μg DNA), incubated at room temperature for 30 min, and then added to the cells. Six hours later, the DNA-transfection reagent mixture was replaced with DMEM containing 10% FBS. After 48 h post-transfection, the expression of the EtMIC2 protein encoded in the transfected plasmid was confirmed by indirect IFA with antibodies against EtMIC2 [7].

### Immunization and parasite-challenge infection

One-day-old chickens were housed in a clean house and at the age of seven days, chickens were randomly selected, weighed, and divided into four groups, each consisting of 20 chickens. Experimental groups were inoculated with 100 μg of either pcDNA3.1(+)-EtMIC2 or empty vector pcDNA3.1(+) by intramuscular injection. Chickens in the challenged control and non-challenged control groups were injected with the same TE buffer at the same site of injection. Seven days after the first vaccination, a booster vaccination was performed as before. Seven days after the booster vaccination, all chickens were infected orally with 10,000 freshly sporulated *E. tenella* oocysts, except for the non-challenged control group, which were inoculated with PBS. All chickens were humanely killed eight days post-challenge to evaluate the lesion score, as previously described [15]. Chicken body weights were measured at day 0 and 8 post-challenge. Feces from each group were collected separately at day 6–8 post-challenge. Oocyst shedding per bird was determined using a McMaster chamber [18, 36]. Each fecal sample was counted three times using the same method. The oocyst decrease ratio (%) was calculated as follows: the average number of oocysts per bird from the challenged control group − the average number of oocysts per bird from vaccinated group/the average number of oocysts per bird from the challenged control group × 100.

### Statistical analysis

All data were subjected to the software SPSS version 20.0 for Windows (IBM, Armonk, NY, USA) for analysis. The Shapiro-Wilk test was performed to assess normality for body weight gains (*p* > 0.05) and oocyst shedding per bird (*p* > 0.05). The significance of differences in body weight gains and oocyst shedding per bird among the groups were evaluated by one-way analysis of variance (ANOVA) and mean values were compared using Duncan’s multiple range tests. The lesion scores were compared by the nonparametric Kruskal–Wallis test. The difference was considered significant if *p* < 0.05.

## Results

### Cloning of the EtMIC2 gene

The EtMIC2 gene was isolated from cDNA of sporulated oocysts from the *E. tenella* Shanghai strain. After cloning and sequencing, a predicted 1127 base pair product was obtained and analyzed with BLASTn. The sequence displayed 100% identity with the known MIC2 gene from the *E. tenella* Beijing strain (GenBank: AF111839.1), suggesting that the EtMIC2 gene from the *E. tenella* Shanghai strain had been successfully amplified. Furthermore, the protein showed a 100%, 99.7% and 99.7% identity with the MIC2 from the *E. tenella* Beijing strain (AAD05559), Houghton strain (XP_013233366.1) and India strain (ACN93990.1), respectively from the GenBank database. Sequence analysis of the EtMIC2 open reading frame identified a polypeptide consisting of 342 amino acid residues with a predicted molecular mass of 35.07 kDa. In addition, the amino acid sequence showed no signs of a transmembrane region; however, it had a signal peptide (residues 1–21).

### Expression and purification of recombinant EtMIC2 protein

**Figure 1:**
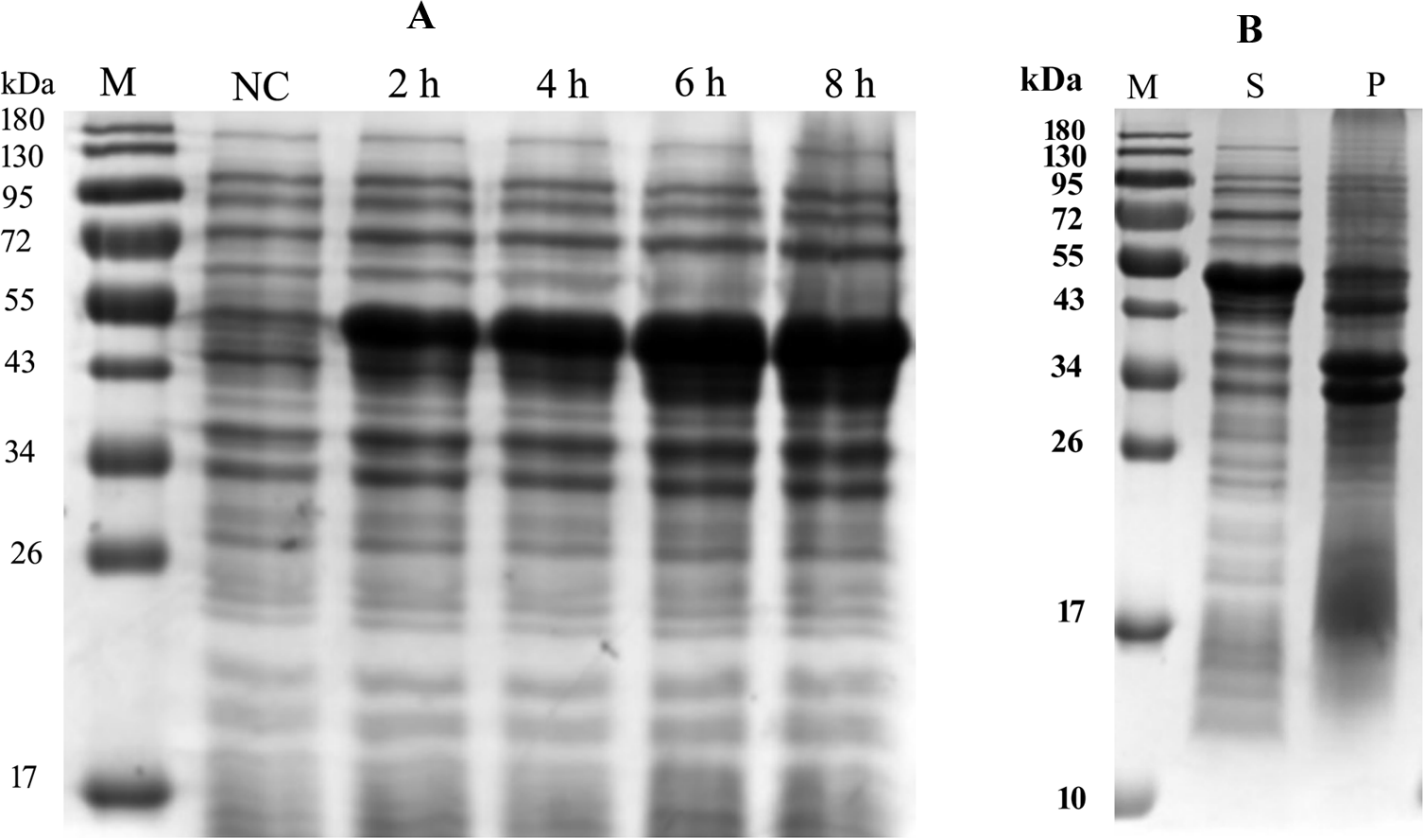
Analysis of EtMIC2 expression in *E. coli* BL21. (A) Protein expression in bacterial pellets at different times of induction. Lane 1 protein marker; Lane 2 negative control (not induced with IPTG); Lanes 3, 4, 5, and 6 induced with IPTG at 2, 4, 6, and 8 h, respectively. (B) Protein expression in sonicated bacterial cells induced with IPTG at 8 h. S: supernatant, P: precipitate.

rEtMIC2 was successfully expressed in both the soluble and insoluble fraction of *E. coli* after induction with 0.8 mM IPTG at 37 °C for 8 h. Purified protein was obtained from the supernatant only using a His Bind Purification kit. The molecular mass of the rEtMIC2 fused to the His-tag was found to be approximately 53 kDa, as expected (Fig. 1).

### Immunofluorescence localization of EtMIC2 in parasites

**Figure 2:**
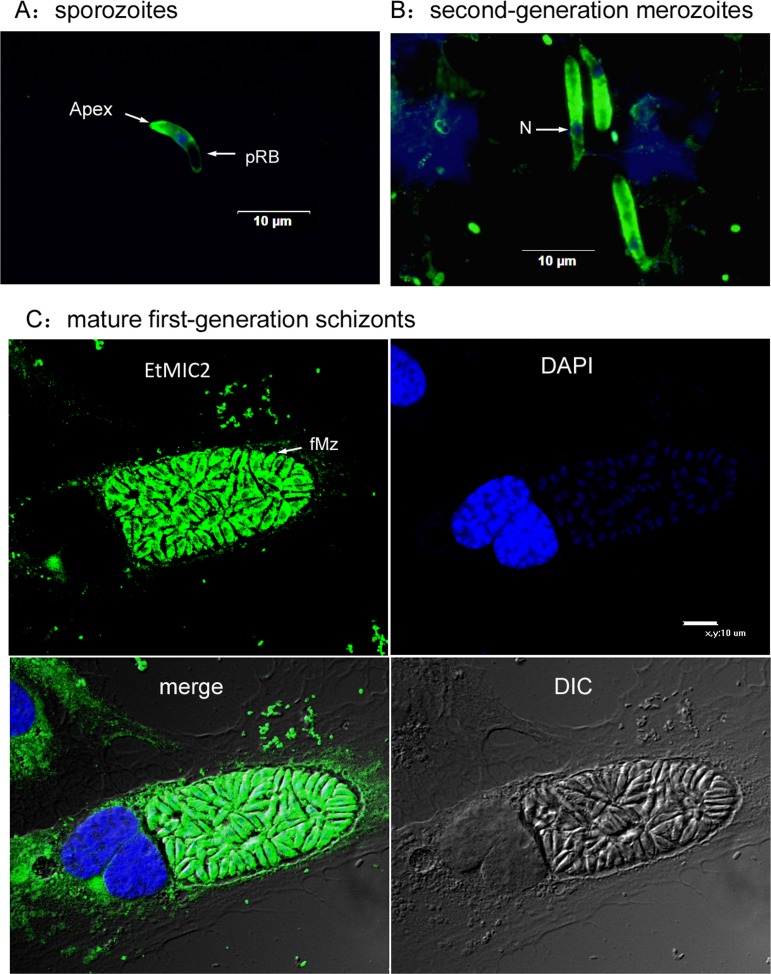
Localization of EtMIC2 in sporozoites, second-generation merozoites, and mature first-generation schizonts by indirect immunofluorescence. The parasites were immunostained with anti-rEtMIC2 antibodies, visualized with FITC (green) and counter-stained with DAPI (blue). Abbreviations: pRB, posterior refractile body; N, nucleus; fMz, first-generation merozoites. *In vitro* sporozoite inhibition assays using recombinant EtMIC2 anti-sera.

The localization of EtMIC2 in sporozoites, second generation merozoites, and mature first-generation schizonts was investigated using an indirect IFA with anti-rEtMIC2 as a probe. Labeled EtMIC2 was found to mainly locate in the anterior region and membrane of newly excysted sporozoites (Fig. 2A). Furthermore, the EtMIC2 protein was found to be strongly expressed in mature first-generation schizonts, and was concentrated in the cytoplasm of first- and second-generation merozoites (Figs. 2B and 2C).

### Inhibition of *E. tenella* invasion by antibodies against rEtMIC2

**Figure 3:**
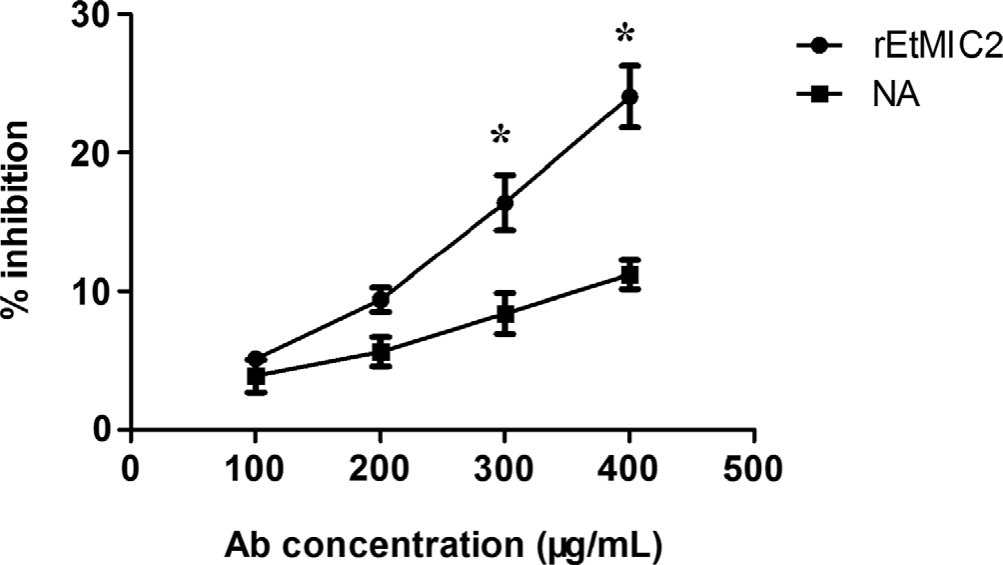
*In vitro* sporozoite inhibition assays using recombinant EtMIC2 anti-sera. Anti-rEtMIC2 stands for IgG purified from recombinant EtMIC2 anti-sera. NA stands for IgG from naive rabbit serum. All assays were performed in triplicate. (*) Indicates that differences between the treatment with anti-rEtMIC2 antibodies and with naive rabbit serum at the same IgG concentration were significant (*p* < 0.05).

An invasion inhibition assay was performed to test the ability of rabbit anti-rEtMIC2 antibodies to inhibit the invasion of DF-1 cells by *E. tenella* sporozoites. It was found that the inhibition effect was in fact dose-dependent (Fig. 3). As compared with the same dose of naive rabbit sera IgG (used as a negative control), pretreatment with 100 or 200 μg/mL anti-EtMIC2 IgG did not significantly affect the invasion capacity of sporozoites (*p* > 0.05); however, pretreatment with 300 and 400 μg/mL did significantly decrease the invasion capacity of *E. tenella* sporozoites (*p* < 0.05).

### Immunofluorescence assay detection of eukaryotic plasmids in DF-1 cells

**Figure 4:**
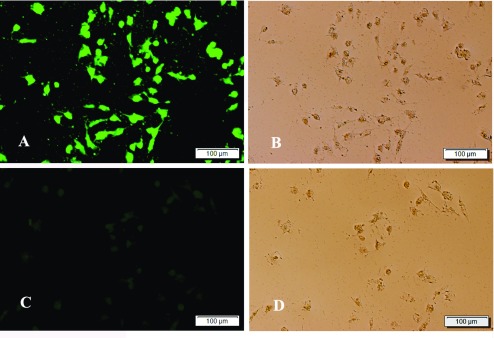
Indirect immunofluorescence assay of EtMIC2 in transfected DF-1 cells. (A, B) pcDNA3.1(+)-EtMIC2-transfected DF-1 cells under fluorescent (FITC) illumination and brightfield, respectively. (C, D) pcDNA3.1(+)-transfected DF-1 cells under fluorescent illumination and brightfield, respectively.

Expression of the recombinant plasmid pcDNA3.1(+)-EtMIC2 and the negative control plasmid pcDNA3.1(+) was confirmed by IFA after transfection with DF-1 cells for 48 h. The intense green fluorescence was detected in DF-1 cells transfected with pcDNA3.1(+)-EtMIC2 and none was in those transfected with pcDNA3.1(+) (Fig. 4), demonstrating that the pcDNA3.1(+)-EtMIC2 protein could be successfully expressed *in vitro*.

### Protective efficacy of pcDNA3.1(+)-EtMIC2 vaccination against *E. tenella* in chickens

The efficacies of the current immunization and challenge assay were evaluated on the basis of body weight gain, oocyst shedding, and the cecal lesion score (Table 1). After challenge, chickens vaccinated with pcDNA3.1(+)-EtMIC2 gained significantly more body weight and had significantly fewer cecal lesions and oocysts compared with chickens vaccinated with the empty vector pcDNA3.1(+) or the TE-challenged controls. Additionally, oocyst shedding in the pcDNA3.1(+) group was significantly lower than in the TE-challenged control groups, but there were no significant differences in terms of body weight and cecal lesions between the two control groups.

**Table 1:** Protective effects of pcDNA3.1(+)-EtMIC2 against experimental *Eimeria tenella* infection in chickens.

Groups	Average body weight gains (g)	Reduced rate of weight gain (%)	Oocyst shedding per bird (10^7^)	Oocyst decrease ratio (%)	Lesion scores
pcDNA3.1(+)-EtMIC2	132.88 ± 38.06^a^	5.44	2.09 ± 0.17^b^	75.30	0.53 ± 0.04^b^
pcDNA3.1(+)	122.61 ± 33.41^b^	12.75	6.17 ± 0.25^c^	27.07	1.54 ± 0.07^c^
TE-infected	118.61 ± 24.40^b^	15.6	8.46 ± 0.24^d^	0	1.58 ± 0.08^c^
TE-uninfected	140.53 ± 26.85^a^	0	0^a^	–	0^a^

Values are expressed as mean ± standard deviation (*SD*). Means in the same column with different letters were found to be significantly different between treatment groups (*p* < 0.05).

## Discussion

In this study, the EtMIC2 gene from *E. tenella* Shanghai strain was cloned and characterized. The obtained sequence showed 99%–100% identity with the available EtMIC2 genes deposited in GenBank, indicating that the EtMIC2 gene is highly conserved among the different strains of *E. tenella* [37]. Furthermore, sequence analysis found that the EtMIC2 gene predicted a protein with a classical signal peptide at the mature N-terminus of the protein. Interestingly, regions of the EtMIC2 protein have previously been found to have highly significant similarities to regions within *Drosophila melanogaster* tropomyosin II and within two substrates of the cellular regulatory enzyme protein kinase C [39].

EtMIC2 is an acidic protein that is abundant within the microneme organelles. Several studies have previously indicated that EtMIC2 forms a complex with EtMIC1, and this complex presumably becomes mobilized from micronemes to the parasite surface during attachment and is then redistributed towards the posterior end of the parasite during penetration of the host cell [39]. Using an antibody raised against the rEtMIC2, immunolocalization studies in *E. tenella* showed that the protein was mainly located in the anterior region and membrane of *E. tenella* sporozoites. Furthermore, the EtMIC2 protein was strongly expressed during first schizogony and was mainly located in the cytoplasm of first- and second-generation merozoites. These results are consistent with previous studies where a monoclonal antibody (mAb) specific to EtMIC2 was used to detect the MIC2 location in several *Eimeria* spp., including *E. tenella*, *E. mitis*, *E. stiedai*, and *E. irresidua*, finding that MIC2 was located in the anterior region of the sporozoites among all *Eimeria* spp. [38]. Additionally, Sasai et al [28] found that the EtMIC2 mAb primarily stained a small region of the apical tip of the sporozoites and merozoites from *E. tenella*, *E. acervulina*, and *E. maxima*. However, there were some differences in the observed staining patterns between the three species. In *E. tenella* and *E. maxima*, there also appeared to be staining of the basal anterior tip [28]. But in *E. acervulina*, a distinct cone-like morphology was stained on the anterior tip of sporozoites and merozoites, with little staining in other regions [28].

Although micronemal in origin, the EtMIC2 protein has been clearly shown to translocate to the surface of the sporozoite, and then during invasion of host cells the protein concentrated around the point of parasite entry, secreted into the extracellular milieu and transferred to the surface of the infected host cell where it stained in uneven patches [39]. These results suggest that EtMIC2 may be involved in cell invasion, but this needs to be confirmed through an invasion assay. Previously, it has been shown that invasion inhibition assays *in vitro* clearly reduced sporozoite invasion in the presence of an mAb or specific polyclonal antibody [12, 13, 24]. In the current study, such assays showed that pretreatments with 300 and 400 μg/mL rEtMIC2-specific antibody, with inhibition rates of 16.41% and 24.06%, respectively, significantly reduced sporozoite penetration of cultured cells. These data therefore confirm that EtMIC2 plays an important role in host invasion.

The lifecycle of *E. tenella* involves endogenous (schizogony and gametogony) and exogenous (sporogony) stages, and the identification of genes expressed in the lifecycle of coccidia is critical in understanding the developmental biology of these parasites. Previously, it has been shown that EtMIC2 is an important microneme protein, expressed abundantly in the sporozoite and schizogony stages [3, 26, 28, 39]. Furthermore, Liu et al. detected the expression of EtMIC2 in the sexual developmental stages of gametocytes and zygotes in chickens artificially infected with *E. tenella* using immunostaining and western blot analysis with a monoclonal anti-EtMIC2 antibody [20]. These results suggest that the protein is actually expressed abundantly in all endogenous developmental stages of *E. tenella* and would be an ideal candidate for vaccine development [47].

Rotational treatment with anticoccidial drugs and a commercial live vaccine is currently the best way to control infection in chicken flocks. Due to the high expense of scaling-up the production of the live parasite vaccine, there have been a number of recent efforts to develop subunit and recombinant coccidiosis vaccines using both DNA and protein-based antigens [16, 21, 30]. However, few have been successful and much more work is needed to identify appropriate antigens and the optimal mode of delivery [17]. The EtMIC2 protein has been found to be conserved in the parasite and its role in the early stages of invasion suggests that it may serve as an effective vaccine antigen. The EtMIC2 gene or rEtMIC2 proteins expressed in prokaryotic, plant or *Pichia pastoris* expression systems have been used as either a DNA vaccine or sub-unit vaccine to immunize chickens against a homologous challenge in several early studies [6, 29, 45, 46]. Taken together, these studies suggest that EtMIC2 can provide partial protection against a challenge, although the protective levels were found to be different between each of these studies. In this study, a recombinant chimeric subunit vaccine was generated, consisting of EtMIC2 and a eukaryotic expression vector, and its efficacy against *E. tenella* infection in chickens was evaluated. The eukaryotic expression vector used in this study pcDNA3.1(+), has been widely used in the development of DNA vaccines against coccidiosis [19, 43, 44]. EtMIC2 protein expression was confirmed with an *in vitro* method before carrying out *in vivo* experiments. Intense fluorescence in DF-1 cells transfected with pcDNA3.1(+)-EtMIC2 indicated that the recombinant plasmid pcDNA3.1(+)-EtMIC2 was successfully constructed and expressed in the eukaryotic cells. Furthermore, the results of the challenge experiments showed that chickens treated with the DNA vaccine gained significantly more weight, and had significantly fewer cecal lesions and oocysts, compared with infected chickens treated with the control vaccine. In the present study, the number of oocysts produced per oocyst administered (the “reproductive potential”) was much lower than that from Williams [42], because a challenge dose higher than the crowding threshold of *E. tenella* was used and parasite replication cannot be accurately assessed due to the crowding effect. In order to minimize this limitation, the relative oocyst decrease ratio [= (oocyst number from challenged unvaccinated group − oocyst number from challenged vaccinated group)/oocysts number from challenged unvaccinated group × 100], instead of the oocyst production, together with weight gains and cecal lesion scores, were used to evaluate the protective efficacy of this DNA vaccine. While these are small-scale experiments, the consistency of the trial and level of efficacy with an approximate reduction of ~75.30% in oocyst shedding following vaccination, being higher than what has been previously seen in many studies with other antigens, indicates that EtMIC2 should be considered as an ideal candidate antigen in the development of a new vaccine against *E. tenella* in chickens.

In summary, EtMIC2 was found to be located in the anterior region and membrane of sporozoites, was strongly expressed during first schizogony, and was mainly located in the cytoplasm of first- and second-generation merozoites. Additionally, rEtMIC2-specific antibody was shown to inhibit parasite invasion. The recombinant plasmid pcDNA3.1(+)-EtMIC2 induced partial protective immunity in immunized chickens. Overall, these results suggest that EtMIC2 may play an important role in parasite cell invasion and may be an ideal candidate for the development of new vaccines against *E. tenella* infection in chickens

## Competing interest

The authors declare that there are no competing interests.
